# Topics of Public Interest

**Published:** 1910-06

**Authors:** 


### TOPICS OF PUBLIC INTEREST.

### Schools in the Open Air

**Buffalo will follow the example of other Cities in this Measure—Conference Held—Superintendent Emerson is in hearty Accord with Project—Plan to be Prepared—Health and School Departments, Physicians, Clergy, Laymen united.**

[*Buffalo Express*, April 6, 1910.]

AS the result of a conference held Tuesday, April 5, in Superintendent Emerson's office, an open-air school for tubercular and otherwise physically defective children, is to be started in Buffalo with as little delay as is consistent with acting wisely, and to Dr. Henry R. Hopkins has been entrusted the chairmanship of a committee, with power to select his own associates, the duties of this committee to consist of drawing up some practical plan of work and submitting it to Mr. Emerson for action.

For a long time the establishment of open-air schools in Buffalo has been considered. Many of the plans suggested were too expensive to be practicable, others were too visionary to be worth consideration, but at Tuesday's meeting, the theories of the theorists, the practical experience of physicians, health department, school department and other active workers were all gathered together, and the result was the appointment of the committee to coöperate with Mr. Emerson, and, if possible, to make the open-air school an immediate part of the educational system of the city.

Superintendent Emerson presided at the hearing and Miss Martha Murray was appointed secretary. Mr. Emerson started the discussion by saying that he had been approached many times by many persons regarding open-air schools, especially for children suffering from tuberculosis in its incipient stages. The trouble, so far, Mr. Emerson said, was that each person wanted a different thing, and he was of the opinion that all interested in the plan would do well to get together and decide upon some general scheme.

### NEED FRESH AIR.

Dr. Henry R. Hopkins was the first speaker, reading a portion of the late Health Commissioner Wende's report for 1906, in which he spoke of the need for such schools, as a preventive measure. According to the sanitary report, there were last June, said Dr. Hopkins, 8,809 defective school children in Buffalo. Of that number more than 5,000 were suffering from other than acute or contagious diseases.

What they all need, said the speaker, is fresh air, and how to give it to them is the problem to be solved. He suggested pavilions on the flat roofs of schoolhouses, or, perhaps, pavilions in the public parks, sightly enough to ornament rather than detract from the beauty of the city's park system.

He spoke of the forest schools of Germany, which have a world-wide reputation, and ended with a stirring appeal to do something in behalf of the physically defective children of Buffalo.

Dr. Jacob Goldberg of the board of school examiners suggested the experiment of trying such a school at No. 44, and said that in finishing No. 1, now being built, it would be a good idea to have the roof made ready for such a purpose. He emphasized the need for fresh-air treatment for the sick children, especially those suffering from hereditary disease or troubles caused by lack of breathing space.

MANY CAN'T BREATHE.

Dr. Hitzel called attention to the fact that ear and throat diseases often make it impossible for the children to get the fresh air when they are in it, and spoke of the trouble the district physicians and school physicians have in getting the people in homes to follow the prescribed treatment for their children. He said that to make such open-air schools popular would do much toward stirring up public sentiment.

Health Commissioner Fronczak said that the health department has been considering many plans for the establishment of such schools and he suggested opening a certain number of schoolrooms in scattered sections of the city, taking out the windows and giving the pupils fresh-air treatment there. He also spoke in approval of the park pavilion plan.

WHY NOT FOREST SCHOOLS?

Dr. Gaertner, while not objecting to open-air schools in the city, was of the opinion that far better results would be obtained by opening such forest schools in the vicinity of Buffalo, but away from the thickly crowded districts. If there are not trees enough near Buffalo for forest schools, set to work and plant them, was his advice. He spoke, too, of the danger from the flying dust and advocated the use of something besides wooden floors in all buildings, especially those in which children are housed.

Dr. F. Park Lewis said that, while he had not given the subject in its present state any great amount of consideration, every movement that would tend toward healthy children was for the betterment of the municipality. "The source of wealth in a community is in its children," said he.

GET BUSY NOW.

Mrs. Henry Altman, who doesn't believe in wasting time over red tape, said that at last week's meeting of the City Federation of Women's Clubs, she presented a resolution, which was adopted, recommending the opening of such a school on one of the city playgrounds, where the only expense entailed would be the erection of a suitable pavilion. She thought that great open-air schools would be a splendid thing, but advocated a small and quick beginning, a practical suggestion that was heartily applauded.

To a question from Superintendent Emerson as to the practicability of taking an upper floor in a schoolhouse, removing the windows and carrying on an open-air school in such a place, Mrs. Edward Kiepe cited her experience while in charge of the tuberculosis classes at Trinity Church. The patients improved rapidly under such conditions, and the windows, which at first she could not persuade them to open more than two or three inches, were in one or two weeks taken out entirely, so that the patients were practically living in the open air.

Dr. Cora B. Lattin, who is to take charge of the women's campaign for better sanitary conditions and better health conditions in Buffalo, urged haste in the opening of the schools, and Miss Bredel instanced the fine work done by Germany, which has 495 institutions for improving the health of the country, especially as it relates to the children.

FIND THE BEST WAY.

Then the Reverend A. V. V. Raymond, D. D., of the First Presbyterian Church, said that the time had gone by when it was necessary to enlist Superintendent Emerson's sympathies, it merely remained to point out the best, most expeditious and economical way of accomplishing what everyone present wanted.

Mr. Emerson said that, judging from the attention he had given the matter, as it has resulted in the foreign schools, he was of the opinion that it had an economic as well as an ethical value, "the true end of all philanthropy," said Dr. Raymond parenthetically, and that it was much better economy to spend a few hundred dollars upon a sickly boy and make him a strong, self-supporting man than to maintain him through life at public expense. He suggested the formation of a committee to prepare a plan and present to him.

It was on this suggestion that Dr. Raymond proposed the name of Dr. Hopkins for chairman of the committee, with power to appoint his own associates. Mr. Emerson asked that Dr. John H. Pryor be put on the committee and to the chairman was left the future plan of campaign, but it was the sense of all present that the move for the establishment of open-air schools in Buffalo has really begun.

### The War Against Tuberculosis

**Dr. William H. Park of New York Declares Tuberculosis Not Due to Infected Milk—Ways to Fight Disease—Colonization in Southwest Advocated—Care of School Children—Discussions in Congress.**

ONLY 2½ per cent. of all tuberculosis in New York City comes from infected milk, butter or meat from bovine sources, according to the statement made by Dr. William H. Park, of that city, May 3, before the pathological section of the National Association for the Study and Prevention of Tuberculosis. Moreover, Dr. Park said, this small percentage is found mainly in children. In other words, pulmonary tuberculosis among adults is contracted solely from human beings, and is not the result of impure milk or foods. Dr. Park supported the contention of Dr. Robert Koch, of Berlin, the discoverer of the tuberculosis germ, when he stood practically alone in declaring that cattle did not transmit pulmonary tuberculosis to human beings. The significance of these conclusions, it was pointed out, will be to direct all the energy of the campaign against tuberculosis to combating the spread of this disease among human beings by preventing spitting, bad housing, overwork and other conditions injurious to health.

Colonisation of consumptives in the Southwest was advocated by Dr. A. M. Forster, of Louisville, before the sociological section. His subject was the best kind of employment for those afflicted with tuberculosis, who when discharged from hospitals and sanatoria relapse into their former condition because of bad environment. Dr. Forster declared that the present machinery in the anti-tuberculosis campaign is inadequate, and did not provide for the sufferer at the most critical time in his existence—when he left the sanatorium or hospital. In view of this fact he advocated that institutions in which tuberculosis cases were treated should be equipped with large farms, on which the convalescents might learn the rudiments of agriculture. He said that in his opinion farming was the most suitable work a cured consumptive could attempt. Dr. Forster therefore advocated that large colonies of consumptives gathered from sanatoria in all parts of the United States should be formed in the Southwest on land which could be irrigated with comparatively little expense, and that here they should live and work for the rest of their lives.

Every city should have one or more outdoor schools, declared Dr. Henry Farnum Stoll, of Hartford, Conn., in a paper read before the sociological section. He recommended such institution for all delicate, socalled scrofulous or anemic children and those with tuberculosis of the bones, who are now in ordinary schools. He also advised the universal use of the skin tuberculin test in the kindergarten and lower grades to detect cases of unrecognised tuberculosis. Dr. Stoll declared that 20 to 40 per cent. of school children in large cities are infected with tuberculosis. This infection usually took place in the home, and not in the school, but the badly ventilated school room, by lowering the child's vitality, allowed the germs to become active. In proof of this statement Dr. Stoll referred to 147 children recently examined by him, in which the question of exposure to tuberculosis in the home was carefully investigated. By the use of tuberculin it was ascertained that 79 per cent. of the children from tuberculous homes were infected, as against only 28 per cent. of those from supposedly healthy homes. It was also found that 50 per cent. of the frail children from healthy homes had the germs of the disease, but that only 13 per cent. of the robust children from similar homes were thus affected.

Dr. S. Adolphus Knopf, of New York, does not believe in too much paternalism on the part of the state to the indigent consumptive, particularly if that paternalism is manifested in the form of a treatment which has not yet received universal recognition as to its efficacy from the medical profession. Neither does he believe in shutting the door in the face of a physician because he has consumption himself or has been closely associated with consumptives. He believes in justice to all consumptives. He presented his views in emphatic language before the clinical section. Incidentally the Legislature of Nebraska and the State Board of Medical Examiners of Oklahoma were severely censured for their attitude toward tuberculosis.

Dr. Park's paper was followed by wide discussion. Dr. Marshall Fabyan, of Boston, presented the story of two cases of human tuberculosis in which the bovine type of bacillus was asserted.

In the clinical section Dr. Albert P. Francine, of Philadelphia, declared that all medical colleges should teach tuberculosis as a special branch and give it a permanent space in the curriculum.

The question of placing the responsibility for relapse of a patient supposedly cured from tuberculosis was discussed following a paper by Dr. W. L. Dunn, of Asheville, N. C., on that subject. Opinion was divided.

An interesting paper was read by Dr. Henry S. Goodall, of New York, who related some of the results he had obtained in the treatment of children for pulmonary tuberculosis. All the experiments detailed were conducted in a sanatorium and were highly successful.

